# SBRT in Lymph-Nodal Oligometastases from Prostate Cancer: Different Outcomes between Pelvic and Para-Aortic Disease

**DOI:** 10.3390/jcm13113291

**Published:** 2024-06-03

**Authors:** Edoardo Pastorello, Luca Nicosia, Luca Triggiani, Francesco Frassine, Paola Vitali, Emiliano Salah El Din Tantawy, Valeria Santoro, Michele Rigo, Simona Gaito, Renzo Mazzarotto, Michela Buglione di Monale e Bastia, Filippo Alongi

**Affiliations:** 1 Advanced Radiation Oncology Department, IRCCS Sacro Cuore Don Calabria Hospital, Cancer Care Center, 37024 Negrar, Italy; edoardo.pastorello@sacrocuore.it (E.P.);; 2 University of Brescia, 25121 Brescia, Italyfrancesco.frassine@gmail.com (F.F.);; 3 Department of Radiation Oncology, Azienda Ospedaliera Universitaria Integrata Verona (AOUI), 37126 Verona, Italy; 4 Department of Clinical Oncology, The Royal Marden Hospital, London SW3 6JJ, UK; 5 Division of Medical Science, The University of Manchester, Manchester M13 9PL, UK

**Keywords:** SBRT, oligometastases, prostate cancer, SABR

## Abstract

**Background**: Lymph-nodal prostate cancer oligometastases are differently treated according to their site: pelvic are locoregional lymph nodes; instead, para-aortic lymph nodes are considered as distant metastases. The aim of the study was a comparison between para-aortic and pelvic oligometastases treated with stereotactic body radiation therapy (SBRT). **Methods**: This is a retrospective analysis. De novo metastatic or extra-nodal disease were excluded. Univariate and multivariate analyses were performed; the pattern of recurrence was also evaluated. A propensity score matching (PSM) was applied to create comparable cohorts. The primary end-point was the progression-free survival (PFS). The secondary end-points were biochemical relapse-free survival (BRFS), ADT-free survival (ADTFS), polymetastases-free survival (PMFS), local progression-free survival (LPFS), and pattern of relapse. **Results**: In total, 240 lymph-nodal oligometastases in 164 patients (127 pelvic and 37 para-aortic) were treated. The median PFS was 20 and 11 months in pelvic and para-aortic patients, respectively (*p* = 0.042). The difference was not confirmed in the multivariate analysis (*p* = 0.06). The median BRFS was 16 and 9 months, respectively, in the pelvic and para-aortic group (*p* = 0.07). No statistically significant differences for ADTFS or PMFS were detected. The cumulative 5-year LPFS was 90.5%. In PSM, no statistically significant differences for all the study end-points were detected. **Conclusions**: Patients affected by para-aortic disease might have a PFS comparable to pelvic disease; local control is high in both cohorts. Our results also support the use of SBRT for para-aortic metastases.

## 1. Introduction

Oligometastatic disease (OMD) is an intermediate state between localized and polymetastatic disease, characterized by a limited number of distant metastases and amenable of local ablative treatment [1,2]. Several factors have been analyzed to identify patients who can benefit more from local treatment, such as the number and size of metastases, the disease-free interval (DFI), and the rate of distant metastases [3,4]. Several OMD subgroups have been identified according to the different onset of metastases, dividing patients with de novo oligometastatic disease from those with a metachronous appearance of metastases, and also separate them according to the response or progression after systemic therapy [5]. Moreover, the histology of the primary tumor is definitely one of the most important factors to evaluate when offering local treatments: for example, prostate cancer (PC) might be characterized in some cases by an indolent clinical behavior with a low tumor burden, relatively slow progression, and, therefore, a good prognosis, which make it a good candidate for local ablative treatments [6,7]. Despite the consideration of OMD and the attempts at characterization, the biological mechanism behind it has not been completely elucidated [8].

In clinical practice, oligometastatic patients are eligible for metastases-directed therapy (MDT), with the aim of improving disease progression, prolonging the use of systemic therapies, or even delaying their start. Stereotactic body radiation therapy (SBRT) as MDT is supported by several trials across different tumor histology and clinical scenarios, demonstrating a high local control level, limited toxicity association, and progression-free survival (PFS) improvement [9,10,11]. The STOMP and ORIOLE trials specifically showed the benefit of SBRT compared to observation in nodal oligometastatic PC patients [12,13]. More recently, the EXTEND trial demonstrated that the addition of SBRT to 6 months of androgen deprivation therapy (ADT) significantly improves PFS, compared to 6 months of ADT alone [14].

The growing use of PET in clinical practice (in particular, choline or prostate-specific membrane antigen—PSMA) has increased the possibility of also detecting oligometastases at low PSA levels [15]. However, in line with the increasing use of PSMA-PET, the problem of its proper clinical use has arisen, because most pivotal studies used conventional imaging; therefore, the comparison with modern PSMA-PET can result in a so-called “migration stage” because of its higher sensitivity. In addition to an earlier identification of oligometastases through PSMA-PET, advancements in radiation therapy technology and its increased accuracy empower clinicians to confidently deliver high-dose treatments [16,17]. 

Lymph nodes are a common site of metastatic spread in PC, exhibiting a characteristic cranial progression along the same nodal station. The spread shows an upward trend, and these nodes are treated differently based on their location: pelvic nodes are classified as locoregional lymph nodes (cN1), while nodes above the aortic bifurcation are considered distant metastases (cM1a). For this reason, treatment choices are influenced by the site of the nodal metastases. Patients with PC nodal metastases generally have a better prognosis than those with bone or visceral metastases [18]. The use of SBRT is well-established in clinical practice for oligometastatic pelvic lymph nodes due to its excellent local control [19]. However, only a few studies have evaluated the use of MDT on para-aortic lymph nodes. We reported the retrospective data of a multi-centric cohort of PC patients who received SBRT for pelvic or para-aortic nodal oligometastases with the aim to compare the clinical outcome.

## 2. Material and Methods

Between 2012 to 2022, a series of oligometastatic PC patients with pelvic or para-aortic lymph-node oligometastases treated with SBRT in 3 different departments (IRCCS Sacro Cuore Don Calabria—Negrar, Brescia University Spedali Civili di Brescia, and Borgo Trento Hospital—Verona) were retrospectively reviewed. This study was approved by the Institutional Review Board and all the data were collected after obtaining informed consent. Patients were included according to the following characteristics: (a) performance status—Eastern Cooperative Oncology Group (PS (ECOG)) ≤ 2; and (b) PC with evidence of pelvic or para-aortic lymph-nodal metastases diagnosed by PSMA or choline PET/CT and treated with SBRT. Patients included in our study had primary controlled malignancy with a number of maximum 5 metastases, with all metastases amenable to SBRT [12]. Patients with de novo synchronous metastatic PC, with history of metastatic disease, or affected by castration-resistant PC were excluded. 

### 2.1. Treatment Procedure

For patients treated with VMAT technique, a simulation CT was acquired in the supine position with a slice thickness of 3 mm. Patients were immobilized with combifix and arms on the chest. When necessary, the diagnostic PET-CT was co-registrered to simulation images to better identify the target. Set-up error was corrected by cone-beam CT before each treatment fraction. The treatment was delivered using the Volumetric Modulated Arc Therapy (VMAT) technique with Flattening Filter-Free modality (FFF). 

For metastases treated with 1.5 T MR-linac, the simulation consisted of a planning CT with a slice thickness of 3 mm, and a 3D T2 weighted MRI with a slice thickness of 1 mm. All scans were acquired with patients in the supine position, with support for the knees and arms on the chest. Planning CT and MR images were rigidly co-registered based on bone anatomy, primarily to obtain bulk densities for each tissue. Target volumes and organs at risk were contoured on an MRI with the aid of co-registered CT and staging diagnostic exams. The same MR scan was performed daily for the treatment procedures. MR-guided treatments were delivered using intensity-modulated radiation therapy (IMRT). In all sessions, the treatment was delivered using adaptive workflow; two possibilities are available: adapt to position (ATP) and adapt to shape (ATS). ATP consists of an online replanning after updating treatment isocenter, matching the current position of target and organs at risk, without recontouring. ATS provides for a complete adaptation of all the contours, followed by a full replanning. Most patients were treated through ATP, which implies a shorter treatment recalculation and can be safely used in the case of no substantial differences in the daily anatomy compared to the reference imaging. Intra-fraction motion was monitored before and during RT administration with a real-time 2D cine-MRI acquired in the coronal and sagittal views [17,20]. 

The gross tumor volume (GTV) was defined as the pathological lymph node and the corresponding PTV was obtained by adding an isotropic expansion of 3 to 5 mm to the GTV. Treatments were prescribed to ensure that at least 95% of the PTV received at least 95% of the prescribed dose, without exceeding 107% at the maximum dose. The median prescription dose was 36 Gy (range 21–45 Gy) in 5 fractions (range 1–6). The constraints for the bowel were V15 Gy, V30 Gy, and V32 Gy < 1 cc in 1, 5, and 6 fractions schedule, respectively.

### 2.2. End-Points and Follow-Up

The primary end-point was the PFS. The secondary end-points were biochemical relapse-free survival (BRFS), local progression-free survival (LPFS), ADT-free survival (ADTFS), and polymetastases-free survival (PMFS). Pattern of recurrence and toxicity were also evaluated. The PFS was defined as the time between SBRT and the radiologic evidence of disease progression (both local or distant). The DPFS was defined as the time between SBRT and the radiological diagnosis of distant progression to new metastatic sites. The BRFS was defined as the time between SBRT and the biochemical relapse, defined as any PSA increase after SBRT. The LPFS was defined as the time between the end of SBRT and the radiological diagnosis of local in-field relapse. The ADTFS was defined as the time between SBRT and the start of ADT. The PMFS was defined as the interval between the SBRT and the onset of more than 3 new metastases. PSA doubling time (PSADT) was calculated using MSKCC algorithm [21]. The DFI was defined as the time between the diagnosis and treatment of the primary tumor and the occurrence of oligometastatic disease. Toxicity was recorded according to the Common Terminology Criteria for Adverse Events (CTCAE version 5.0) as acute (within 60 days from SBRT end) and late (more than 60 days after SRT end). PSA monitoring was conducted every 3 months in the first 2 years after SBRT and every 6 months later; in the case of an increase in PSA levels following SBRT, 18F-PSMA PET-TC imaging was conducted. 

### 2.3. Statistical Analysis

Survival analysis was performed using the Kaplan–Meier method. The univariate analysis was performed using the log-rank test and the multivariate analysis was performed with Cox proportional hazard regression including all the relevant covariates in the univariate analysis (*p* ≤ 0.10). In particular, the following covariates were evaluated: age, initial PSA, class risk, ISUP score, PSA at SBRT, PET used (choline or PSMA), number of lymph nodes, RT dose, number of fractions, previous ADT, association of ADT, PSA DT, DFI, and treatment on primitive site. The Chi-squared test in 2 × 2 contingency tables was used to evaluate the pattern of relapse. A propensity-score-matched (PSM) analysis was performed by matching patients with pelvic or para-aortic lymph nodes in a 1:1 ratio, based on the following four covariates: PSA before SBRT (allowed variation +/−0.5 ng/mL); DFI (under or over 72.5 months); number of treated lymph nodes (one versus more than one); and risk class at diagnosis (low, intermediate, or high risk). A further survival analysis on the new obtained groups was conducted. The statistical analysis was performed through the software SPSS v.22 (IBM software, Armonk, NY, USA). A *p* value < 0.05 was indicative of a statistically significant correlation.

## 3. Results

From 2012 to 2022, 164 PC patients were treated with SBRT. One-hundred twenty-seven (127) patients had pelvic oligometastases and thirty-seven (37) had para-aortic oligometastases. The baseline characteristics were balanced between the groups. The median age was 71 years for both treatment groups (*p* = 0.65). The median PSA before SBRT administration was 1.16 ng/mL and 1.33 ng/mL for pelvic and para-aortic patients, respectively (*p* = 0.48). Forty-three (43) (39.4%) patients and twenty (20) (54%) have received ADT before SBRT (*p* = 0.026) as a part of a curative treatment for the primary tumor, alongside prostate radiotherapy during salvage RT to the prostatic bed. The patients’ characteristics for both treatment groups are summarized in Table 1. 

### 3.1. Progression-Free Survival

**Figure 1:**
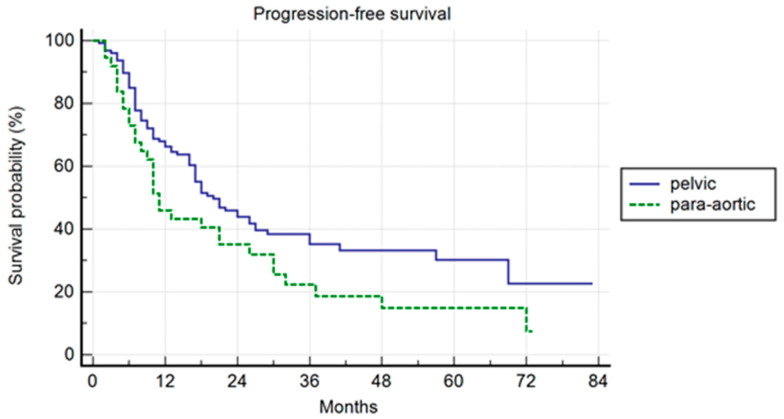
Kaplan–Meier curve showing progression-free survival (PFS) in the entire population stratified by lymphnode metastases site (pelvic versus para-aortic) (*p* = 0.06; HR 1.49; IC: 0.98–2.28).

The median follow-up was 37 months. Pelvic patients had a 1-, 3-, and 5-year PFS of 66.3%, 35.2%, and 30.2%, respectively, with a median PFS of 20 months (range 2–80). Para-aortic group had a 1-, 3-, and 5-year PFS of 45.9%, 22.4%, and 14.9%, respectively, with a median PFS of 11 months (range 2–73 (*p* = 0.042)) (Figure 1). The difference was not confirmed in the multivariate analysis, but a trend was maintained (*p* = 0.06; HR 1.49; IC: 0.98–2.28). In the multivariate analysis, the association of ADT correlated with a longer PFS (*p* = 0.04; HR 0.49; IC: 0.25–0.98). In the uni- and multivariate analysis, the lower PSA value at SBRT correlated with a longer PFS (*p* = 0.03 and *p* = 0.04; HR: 1.47: IC: 1.01–2.28) (Table 2).

### 3.2. Biochemical Relapse-Free Survival

**Figure 2:**
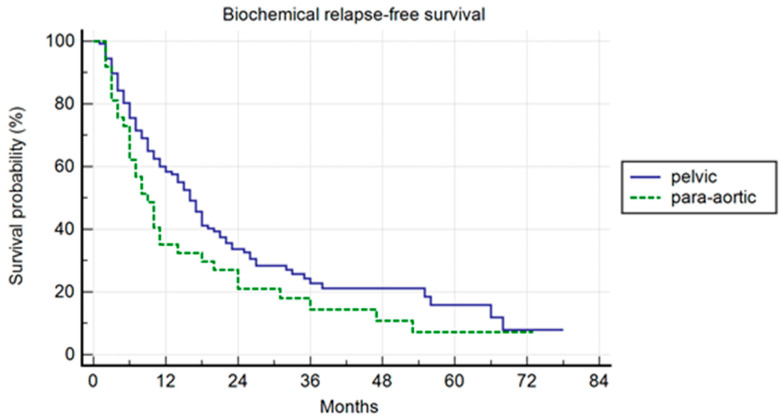
Kaplan–Meier curve showing biochemical relapse-free survival (BRFS) in the entire population stratified by lymph node metastases site (pelvic versus para-aortic) (*p* = 0.01; HR: 0.47; IC: 0.25–0.87).

Pelvic patients had a 1-, 3-, and 5-years BRFS of 59.4%, 22.8%, and 15.9%, with a median BRFS of 16 months (range 1–78); the para-aortic group had a 1-, 3-, and 5-year BRFS of 35.1%, 14.4%, and 7.2%, with a median BRFS of 9 months (range 2–73) (*p* = 0.007) (Figure 2). In the univariate and multivariate analysis, the association of ADT to SBRT correlated with a longer BRFS (*p* = 0.01; HR: 0.47; IC: 0.25–0.87).

### 3.3. ADT-Free Survival

The subgroup of patients with pelvic lymph-node involvement had a 1-, 3-, and 5-year ADTFS of 73.2%, 38.3%, and 25.4%, respectively, with a median ADTFS of 28 months (range 2–84), whilst the para-aortic group had a 1-, 3-, and 5-year ADTFS of 64.9%, 39.2%, and 19.6%, respectively, with a median ADTFS of 19 months (3–78) (*p* = 0.43). In the univariate and multivariate analysis, the number of treated lymph nodes was associated with ADTFS: patients treated at one lymph node had a better ADTFS than patients treated at more than one lymph node (*p* = 0.001; HR 1.68; IC: 1.08–2.59).

### 3.4. Polymetastatic-Free Survival

Pelvic patients had a 1-, 3-, and 5-year PMFS of 94.3%, 82.9%, and 75.4%; para-aortic patients had a 1-, 3-, and 5-year PMFS, respectively, of 94.3%, 82.9%, and 62.4% (*p* = 0.10). Both in the univariate and multivariate analysis, the number of treated nodes was associated with PMFS (*p* = 0.03; HR 2.01; IC: 1.06–3.78). The 5-year overall local control was 90.5%.

### 3.5. Pattern of Relapse

The pattern of relapse was also evaluated. Forty (40) pelvic patients recurred in the pelvis again (51.8%), while only four (4) para-aortic patients (12.9%) had a pelvic relapse (*p* = 0.001). Para-aortic patients relapsed more on para-aortic lymph nodes (Table 3). At first progression, 77% pelvic and 71% para-aortic patients maintained an oligometastatic disease (*p* = 0.50). No toxicity events of grade 2 or higher were recorded.

### 3.6. Propensity Score Analysis

**Figure 3:**
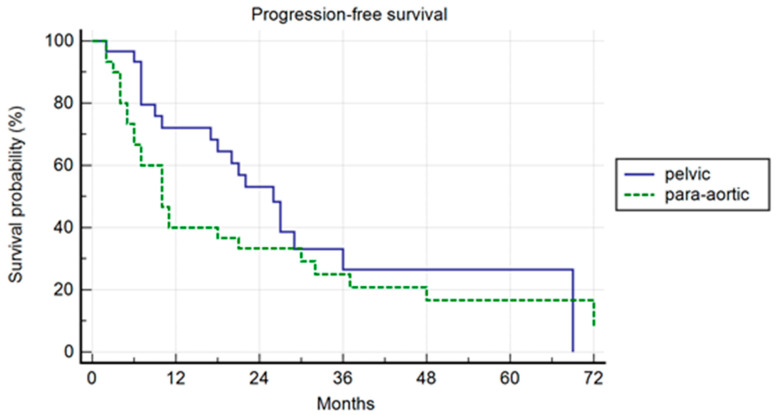
Kaplan–Meier curve showing progression-free survival (PFS) in the matched population stratified by lymphnode metastases site (pelvic versus para-aortic) (*p* = 0.20).

**Figure 4:**
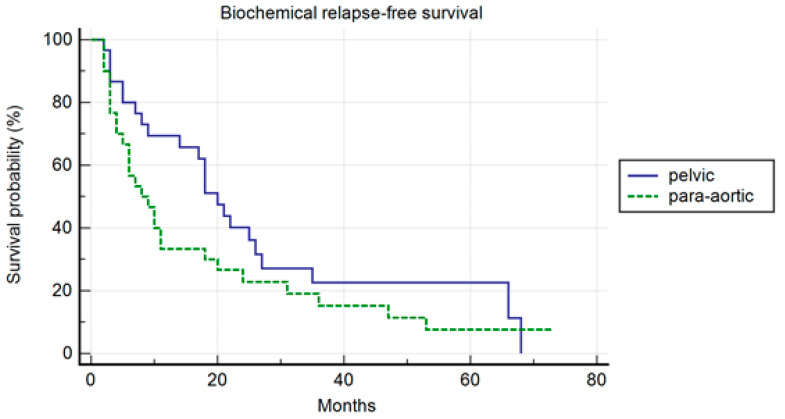
Kaplan–Meier curve biochemical relapse-free survival (BRFS) in the matched population stratified by lymphnode metastases site (pelvic versus para-aortic) (*p* = 0.14).

In the PSM analysis, randomization was 1:1, and 30 patients for each cohort were compared. The patients’ characteristics are summarized in Table 4. The median PFS was 26 months and 10 months, respectively, in the pelvic and para-aortic group (*p* = 0.20) (Figure 3). One-, three-, and five-year PFS were 72.1%, 26%.5, and 26.5% in pelvic patients and 40%, 25%, and 16.7% in para-aortic patients. The median BRFS was 12 months and 8 months, respectively, for pelvic and para-aortic patients (*p* = 0.14) (Figure 4). One-, three-, and five-year PFS were 69.4%, 22.6%, and 22.6%, in pelvic patients and 33.3%, 15.2%, and 7.6%. in para-aortic patients. The median ADTFS was 28 months and 19 months, respectively, for pelvic and para-aortic patients (*p* = 0.67).

No statistical differences were recorded between the two groups in CRFS, PMFS, and LPFS.

## 4. Discussion

Modern radiotherapy enables the precise delivery of high-dose treatments with minimal toxicity. SBRT is widely accepted as an effective treatment for oligometastases and PC patients may benefit from MDT in both hormone-sensitive and castration-resistant settings. Patients affected by nodal-exclusive OMD are typically characterized by a more indolent disease than those affected by polymetastatic or visceral metastases: in this setting, local treatments, such as SBRT, are able to delay the start of systemic therapy, prolong its duration, or postpone the switch to a subsequent treatment line [19,22,23,24].

In the present study, we compared the efficacy of SBRT in patients affected by PC with pelvic and para-aortic oligometastases. Para-aortic patients showed similar outcomes to pelvic patients. Specifically, we found no statistically significant difference in PFS or BRFS. This observation can be explained by the low number of patients with para-aortic oligometastases and by the different features at the time of diagnosis of metastatic disease. Patients with para-aortic disease presented more frequently with >1 pathological lymph nodes compared to pelvic patients that more frequently had a single nodal site. Despite the higher proportion of patients with unfavourable clinical variables in the para-aortic group, no statistically significant differences in PFS or BRFS were observed, thus suggesting that SBRT might also be considered a valid treatment option in patients affected by para-aortic lymph nodes.

The median PFS in our study was in line with the literature data on nodal oligometastatic patients treated with SBRT: 14 months in the ORIOLE study and 21 months in the STOMP trial [12,13]. However, we must consider the different staging techniques used: the STOMP trial included choline PET staged patients, and the ORIOLE trial was based mainly on a CT and bone scan, while, in our study, the diagnosis was made through PET (choline or PSMA) to all patients. The use of PET allows the early diagnosis of oligometastatic disease compared to conventional imaging. This is particularly true for nodal oligometastases, that are typically small in the oligometastatic setting, not detectable by bone scan and hard to define with a CT. Nodal oligometastatic PC represents a more indolent disease subtype that can potentially benefit more from local ablative treatments. A study by Ost et al. documented that patients with nodal oligometastases typically continue to experience recurrences in the lymph nodes. In particular, pelvic oligometastatic patients frequently recur, whereas only one-third of para-aortic patients eventually relapse in the lymph-nodal station [25]. 

More recently, Francolini et al. evaluated the pattern of relapse after SBRT in nodal para-aortic oligometastatic PC patients. The PFS was 10 months and 43% of recurrences were in the para-aortic nodal station [26]. Those data are comparable to our cohort, although, in the abovementioned study, both hormone-sensitive and castration-resistant PC patients were included.

Rich et al. described a different approach for treating para-aortic oligometastases by including the entire para-aortic nodal station with a dose of 45–50 Gy and a simultaneous integrated boost up to 60–65 Gy in PET-positive nodes in a series of 34 PC patients [27]. In this study, the 2-year PFS was 83.4%, but two G3 events (one gastrointestinal and one urinary) were recorded. Interestingly, the PFS was higher compared to our study. As previously described, in our population, 38.7% of para-aortic patients had a new oligorecurrence cranially in the same nodal station, suggesting the presence of a microscopic disease at the time of SBRT in this patient proportion. Considering this, the inclusion of a larger prophylactic volume might improve disease control. However, while, on one hand, this approach might lead to a higher toxicity [27], on the other hand, SBRT to nodal oligometastases in PC is virtually free from severe side effects and can be safely administered in metachronous oligometastases [12,13,14]. In fact, in this cohort, more than half of the relapses occurred at other sites. For these patients with multiple microscopic metastases, a larger treatment volume would not have improved their prognosis. In addition, in our study, more than 70% of patients had a sequential oligoprogression potentially amenable to an SBRT rechallenge, suggesting that PFS may not the best end-point to compare different radiotherapy approaches. No differences in the rate of metastatic progression between pelvic and para-aortic nodes were found in our study. Considering the relatively indolent clinical behavior of the para-aortic lymph node and the possibility of effectively treating those lesions with SBRT, in the OMD disease spectrum, this localization could be considered closer to the pelvic lymph-node spread than a purely distant metastatic disease. Therefore, a focused approach with a combination of SBRT with or without systemic treatment might be considered in selected cases.

This study, however, is not free from limitations: its retrospective nature exposes it to potential bias, though partly mitigated by the PSM analysis, and there is a limited number of para-aortic patients, which may have reduced the statistical power. Lastly, the different use of ADT between groups and the incomplete information regarding previous pelvic nodal dissection might have had an impact on survival end-points. The definition of a specific setting of metastatic PC and diagnosis through PSMA or choline PET for all metastases represent instead a potential strength. Currently, there is ongoing research to identify patients with pure oligometastatic disease and determine the relevant characteristics that can predict their response to MDT. Emerging evidence seems to show that PC with exclusive lymph-node metastases can be considered a subset of metastatic PC with a peculiar indolent behavior and amenable to MDT. Definitely, in the future, genomic profiling will also help us to identify patients that can benefit the most from SBRT in the treatment of oligometastases.

## 5. Conclusions

Oligometastatic para-aortic disease seems to have similar outcomes compared to pelvic disease in selected patients. Both groups have shown good local control with no relevant toxicity. Our data support the use of SBRT for para-aortic oligometastases with the aim of postponing systemic therapy or prolonging its use. Further randomized prospective trials are warranted, to provide supporting evidence for SBRT administration in this setting and to elucidate which patients could benefit the most from this approach.

## Figures and Tables

**Table 1:** Patients’ characteristics.

	Pelvic (127)	Para-Aortic (37)	*p*
Age (median)	71 (range 56–89)	71 (59–86)	0.65
Initial PSA (ng/mL)	8.4 (1.3–86)	10.9 (3.5–50)	0.76
Risk class at diagnosis	Low: 12 (9.4%)	Low: 2 (5.4%)	0.4
	Intermediate: 30 (23.6%)	Intermediate: 6 (16.2%)	
	High: 85 (66.9%)	High: 29 (78.4%)	
ISUP score	1: 15 (11.8%)	1: 3 (8.1%)	0.56
	2: 12 (9.4%)	2: 3 (8.1%)	
	3: 33 (26%)	3: 7 (18.9%)	
	4: 36 (28.3%)	4: 14 (37.8%)	
	5: 31 (24.4%)	5: 10 (27%)	
First treatment on primary site	Surgery: 109 (85.8%) RT: 18 (14.2%)	Surgery: 30 (81.1%) RT: 7 (18.9%)	0.48
Salvage RT	80 (73.4%)	25 (83.3%)	0.61
Previous pelvic treatments (LAD or pelvic RT)	60 (47.2%)	22 (59.4%)	0.19
Previous ADT	43 (39.4%)	20 (54%)	0.026
PSA at SBRT (ng/mL)	1.16 (0.1–16.4)	1.33 (0.23–5.25)	0.48
PSA DT (months)	4.7 (1–17)	5.25 (1–17)	0.84
DFI (months)	69 (3–246)	78 (6–223)	0.98
PET	choline 62 (48.8%) PSMA 65 (51.2%)	choline 15 (40.5%) PSMA 22 (59.5%)	0.37
Number of treated lymph nodes (median)	1 (1–3)	1 (1–3)	/
More than 1 lymph nodes treated	35 (27.6%)	18 (48.6%)	0.016
Median RT dose (Gy)	36 (21–45)	36 (21–45)	0.40
Number of fractions	5 (1–6)	5 (1–6)	0.21
RT technique	VMAT 112 (88.2%) MR Linac 15 (11.8%)	VMAT 33 (89.2%) MR Linac 4 (10.8%)	0.86
Concurrent ADT	13 (10.2%)	5 (13.5%)	0.57

RT: radiotherapy; LAD: lymphadenectomy; ADT: androgen deprivation therapy; SBRT: stereotactic body radiotherapy; DT: doubling time; DFI: disease-free interval; VMAT: volumetric modulated arc therapy.

**Table 2:** PFS at univariate and multivariate analysis.

	Univariate	Multivariate
PSA at SBRT	*p* = 0.036	*p* = 0.04 (HR: 1.47; IC: 1.01–2.28)
PSA DT	*p* = 0.31	/
DFI	*p* = 0.79	/
PET choline vs. PSMA	*p* = 0.25	/
Number of lymph nodes treated (1 vs. >1)	*p* = 0.14	/
Previous pelvic treatments	*p* = 0.67	/
Previous ADT	*p* = 0.17	/
Concurrent ADT	*p* = 0.058	*p* = 0.04 (HR 0.49; IC: 0.25–0.98)
Treatment on primitive site (surgery vs. RT)	*p* = 0.26	/
Pelvic vs. para-aortic	*p* = 0.042	*p* = 0.06 (HR: 1.49; IC: 0.98–2.28)

SBRT: stereotactic body radiotherapy; DT: doubling time; DFI: disease-free interval; ADT: androgen deprivation therapy; RT: radiotherapy.

**Table 3:** Pattern of relapse.

Site of Relapse	Total	Pelvic	Para-Aortic	*p*
Prostate bed	1	1 (1.3%)	0	/
Pelvis	44	40 (51.9%)	4 (12.9%)	0.001
Para-aortic nodes	25	13 (16.9%)	12 (38.7%)	0.16
Other lymph nodes	6	3 (3.9%)	3 (9.6%)	0.24
Metastases	32	20 (26%)	12 (38.7%)	0.2
Total	108	77	31	

**Table 4:** PSM: Patients’ characteristics.

	Pelvic (30)	Para-Aortic (30)	*p*
High-risk PC	26	26	1
PSA at SBRT	1.09 ng/mL (0.17–4.77)	1.1 ng/mL (0.23–4.67)	0.6
Median DFI	78.5 (11–155)	79 mesi (11–223)	1
SBRT on 1 node	17	17	1
Surgery as first treatment	26	26	1
Previous ADT	15	17	0.53
PSA DT months	3.5 (2–8)	4.5 (1.5–17)	0.24
Concurrent ADT	4	3	0.69

PC: prostate cancer; SBRT: stereotactic body radiotherapy; DFI: disease-free interval; ADT: androgen deprivation therapy; DT: doubling time.

## Data Availability

The data presented in this study are available on request from the corresponding author.
